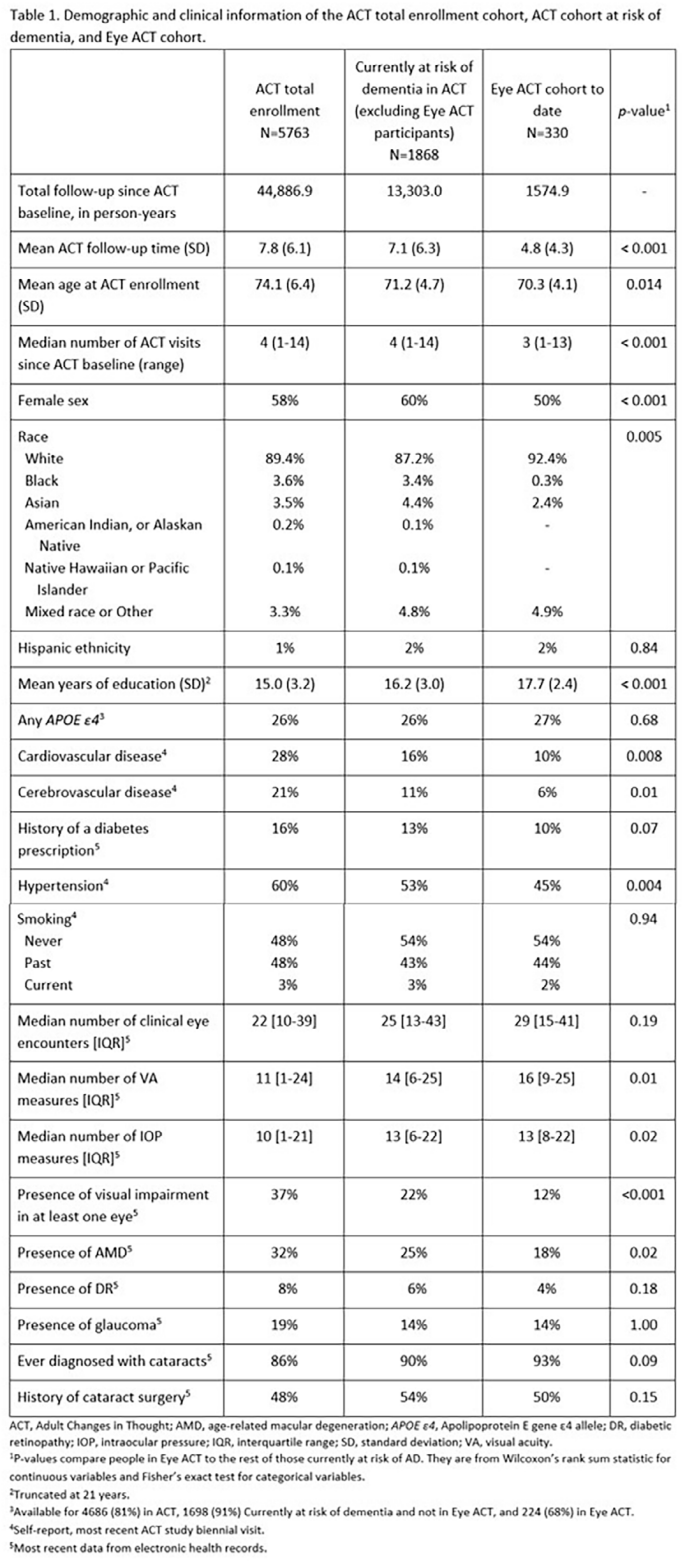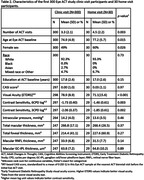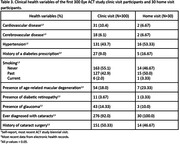# Eye Adult Changes in Thought (Eye ACT) study: Settings and report on the inaugural cohort

**DOI:** 10.1002/alz.083941

**Published:** 2025-01-09

**Authors:** Alina N Ferguson, Missy Takahashi, Beth Pope, Beverly Schaaf, Julie Cooper, Jason Kam, Michael Brush, Laura E. Gibbons, Aaron Y Lee, David Arteburn, Eric B Larson, Paul K. Crane, Cecilia S Lee, the Eye ACT study

**Affiliations:** ^1^ Department of Ophthalmology, University of Washington, Seattle, WA USA; ^2^ The Roger and Angie Karalis Johnson Retina Center, Seattle, WA USA; ^3^ University of Washington School of Medicine, Seattle, WA USA; ^4^ Kaiser Permanente Washington Health Research Institute, Seattle, WA USA; ^5^ Kaiser Permanente Washington, Seattle, WA USA; ^6^ Department of General Internal Medicine, University of Washington, Seattle, WA USA

## Abstract

**Background:**

To describe the settings and compare demographic and baseline clinical factors of the inaugural Eye Adult Changes in Thought (ACT) study participants.

**Method:**

Adult Changes in Thought (ACT) is an ongoing cohort study of older adults (≥ 65 years) randomly recruited from Kaiser Permanente Washington who were cognitively normal at enrollment and followed biennially for the onset of Alzheimer’s disease since 1994. Cognitive testing included the Cognitive Abilities Screening Instrument scored using Item Response Theory (CASI‐IRT) with other measures of cognition. Since December 2021, the Eye ACT study has recruited from the parent ACT study and assessed visual function, intraocular pressure, and multimodal retinal imaging. Eye ACT is unique because participants may be evaluated at the research clinic or in their homes. We compared demographic and clinical characteristics of Eye ACT versus parent ACT participants, and within Eye ACT, participants seen in research clinics versus those seen at home.

**Result:**

Compared to current ACT participants (N=1868), Eye ACT participants (N=330) were younger, newer to ACT, and had more years of education at ACT enrollment. At their latest assessment, Eye ACT participants reported better cardiovascular health (lower rates of cardiovascular disease, cerebrovascular disease, and hypertension, *p*‐values all <0.05) and had lower rates of visual impairment (12% vs. 22%, *p*<0.001) and age‐related macular degeneration (18% vs. 25%, *p*=0.02). At their first Eye ACT visit, people seen at home (N=30) were older (mean age 77.2 vs. 74.9, *p*=0.015), more likely to be female (60% vs. 49%, *p*=0.026), and had significantly worse visual acuity (71.1 vs. 78.9 Early Treatment Diabetic Retinopathy Study [ETDRS] letters, *p*<0.001) and contrast sensitivity (‐1.85 vs. ‐2.06 mean log units at 3 cycles per degree [CPD], *p*=0.002) compared to those seen in clinic (N=300). CASI‐IRT scores and optical coherence measurements were similar between the two groups.

**Conclusion:**

Eye ACT home visit participants had significantly decreased visual function measures compared to research clinic participants. Home visits enable data collection from participants who are often underrepresented in dementia research and may have different dementia risk profiles. Functional measurements of retinal health and study settings are important considerations when evaluating potential retinal biomarkers of dementia.